# Accessing 3D Location of Standing Pelvis: Relative Position of Sacral Plateau and Acetabular Cavities versus Pelvis

**DOI:** 10.1155/2012/685497

**Published:** 2012-04-10

**Authors:** E. Berthonnaud, R. Hilmi, J. Dimnet

**Affiliations:** ^1^L'Hôpital Nord Ouest, Site de Villefranche/Saône, BP 436, 69655 Villefranche/Saône Cedex, France; ^2^Laboratoire de Physiologie de l'Exercice, Université de Lyon, 42023 Saint Etienne, France; ^3^Group of Applied Research in Orthopaedic, 69005 Lyon, France

## Abstract

The goal of this paper is to access to pelvis position and morphology in standing posture and to determine the relative locations of their articular surfaces. This is obtained from coupling biplanar radiography and bone modeling. The technique involves different successive steps. Punctual landmarks are first reconstructed, in space, from their projected images, identified on two orthogonal standing X-rays. Geometric models, of global pelvis and articular surfaces, are determined from punctual landmarks. The global pelvis is represented as a triangle of summits: the two femoral head centers and the sacral plateau center. The two acetabular cavities are modeled as hemispheres. The anterior sacral plateau edge is represented by an hemi-ellipsis. The modeled articular surfaces are projected on each X-ray. Their optimal location is obtained when the projected contours of their models best fit real outlines identified from landmark images. Linear and angular parameters characterizing the position of global pelvis and articular surfaces are calculated from the corresponding sets of axis. Relative positions of sacral plateau, and acetabular cavities, are then calculated. Two hundred standing pelvis, of subjects and scoliotic patients, have been studied. Examples are presented. They focus upon pelvis orientations, relative positions of articular surfaces, and pelvis asymmetries.

## 1. Introduction

Numerous studies described pelvis patient morphology, including, sometimes, relative positions of acetabular cavities, because of recent developments of total hip arthroplasty. Direct measurements showed morphometrical standing pelvis features [1]. 3D computed tomography has been applied to different positions of pelvis for accessing flexion angle [2]. A registered bone atlas has been used, for best fitting pelvis position and for localizing the corresponding coordinate system [3].

A new radiographic system, with simultaneous frontal and sagittal exposures, has been recently set in radiologic departments [4]. Bone punctual landmarks are reconstructed, in space, from their projected images, a registered bone model is projected on both X-rays, and its shapes are slightly altered by the experimenter, in order to fit bone landmark projections.

In case of total hip arthroplasty, a cup is fixed in the acetabular cavity and a femoral component replaces the upper femoral head and neck. Orientations of acetabular and femoral implant components influence hip range of motion [5]. Effects of different cup orientations in total hip arthroplasty have been simulated [6]. During total hip arthroplasty, the acetabular cavity orientation may be estimated from the treatment of the motion cone, corresponding to a patient thigh circumduction movement, observed by cameras [7]. Relative position of acetabular cavity, versus pelvis, is registered in preoperative situation and used during surgical operation [8, 9].

Since a long time ago, our group of research is implicated in clinical applications of biplanar radiographic examination of patients with successive exposures. The technique involves only a standard radiographic set up, with one X-ray plate and one X-ray source, and an interposed rotating plateau, upon which the patient stands motionless [10]. In standard photogrammetry, a point is reconstructed in space from its two X-ray images. Two rays, connecting X-ray sources to projected images, intersect together at the level of the unknown point. In real situation, the two rays intersect rarely. The point is located along the common perpendicular to the two projecting rays. A calibration procedure, involving the use of a calibration object, alters slightly the X-ray source position, in order to minimize linear offsets between rays. The photogrammetric technique must be mended for clinical applications. Generally, bone punctual landmarks have not two X-ray clear images. The technique of epipolar planes [11] is applied to improve the location of blurry projections. When successively radiographed, patients may move between exposures. An autocalibration procedure is applied. Some leading punctual landmarks are identifiable on both X-rays. Positions of X-ray sources are altered in order to minimize offsets between all couples of rays. Reconstructed punctual landmarks are then projected radiographically on each X-ray. Offsets between real images and landmark projections allow experimenter to estimate reconstruction errors. They have been calculated for each clinical application (±1 mm for 3D punctual landmarks and ±1° for reconstructed bone axes [12]).

The biplanar radiographic technique, with successive exposures, has been only applied for reconstructing bone punctual landmarks and bone axes, in standing patients. Bone skeletons are defined. They link together bone anatomical centers and axes. For each patient, bone skeletons characterize the corresponding personalized bone structure [13]. A set of axis is affixed to each bone skeleton. The standing bone frame location, versus the fixed radiologic frame, determines bone linear position and angular orientation. This technique has been applied to long bones of lower limb: femurs, tibia, fibula [14], and to bones of shoulder complex [15]. The method has been tested with deformable bone structures: standing asymptomatic and scoliotic spines. The spinal curve, passing through vertebral body centroids, is reconstructed in space from points extracted from frontal and sagittal X-rays. Spinal geometric structures are then determined. Temporal evolutions of spinal structures have been applied to study evolutive deforming pathologies [16].

This paper presents an application of biplanar radiography, to the global pelvis, including its articular surfaces. However, locating articular surface needs the proposal of new techniques. The global pelvis is directly located, in space, from three reconstructed leading points: the femoral head centers and the sacral plateau center. The triangle drawn from these points is the pelvic skeleton. The pelvic frame is defined from this skeleton.

The access to articular surfaces is different. They are first modeled geometrically: acetabular cavities are represented as hemispheres, the sacral plateau is a plane, bounded anteriorly by a hemiellipsis. Frontal and sagittal projections of circular acetabular cavity brims and sacral plateau outlines have elliptic shapes. Brims of the modeled articular surfaces are projected radiographically on the two views. Their projections are best fitted to the real outlines. Local sets of axis are affixed to optimal location of articular surface models. Set of axis positions gives pelvic global orientation and relative position of articular surfaces.

Several examples are presented and discussed. They focus on very different pelvis and articular surfaces features and orientations, and on pelvic asymmetries, coupled or not with back deformities.

## 2. Material

Two hundred standing biplanar radiographic files of asymptomatic subjects (100) and scoliotic patients (100) have been recorded and treated, in the frame of pelvis/spine studies. Radiographic examinations involved frontal and sagittal exposures grasped successively.

A standard radiographic set up is used, involving a rotating platform, interposed between radiographic source and plate. Platform orientations at 0° (frontal view) and at 90° (sagittal view) are determined owing to a mechanical locking system. Positions of experimental set up: X-ray source, X-ray plate, and rotating platform, are refined from applying a calibration procedure [15], including the use of an object of calibration. Patients must stand motionless on the platform. Platform and motionless patient are moved for two successive grasps. Bearing poles help patients to keep a stable posture. Two numerical radiographs (sizes 30 cm × 90 cm) are shot. A self-calibration procedure is then applied to the two radiographs. It allows taking in account small patient movements occurring between successive grasps. The self-calibration technique is based upon epipolar plane geometric properties.

## 3. Methods

In previous studies, the biplanar radiographic technique with successive exposures has been only applied to the 3D reconstruction of points and anatomical axes from their X-ray images. Sets of bone punctual landmarks and axes have been linked together for defining bone skeletons. Each standing bone skeleton is considered as representing its personalized geometric model. A bone frame is defined from the bone skeleton.

In the present study, a same technique is used for the global pelvis modeling. But, in case of pelvis articular surfaces, the biplanar radiographic approach must be completed from fitting projected models to real contour images. The conical projecting operator must be strictly analogous to the radiologic process: same locations of X-ray plate and punctual X-source. The quality of conical projections depends highly on accurate location of X-ray source and plate. This is obtained through using a calibration technique, involving a calibration object followed by an autocalibration procedure applied to each subject or patient.

### 3.1. The Pelvis (Figure 1)

A simplified model of pelvis had been defined for clinical studies. The pelvis was represented by three points: the two femoral head centers and the sacral plate center. Femoral head center projections are easily detected on frontal and sagittal X-rays. Frontal projection of the anterior part of the sacral plateau may be modeled by a hemiellipsis of which center is calculated. The blurry sagittal projection of this center is refined using epipolar plane properties. The 3D position of the sacral plateau center is defined as the point where rays connecting X-ray sources to projected center images intersect. Femoral head center and sacral plateau center are the three summits of the pelvis triangle. The pelvis set of axis is defined from this triangle. The pelvis frame is centered at the mid-point *O*_*p*_ of the femoral head centers. The femoral head axis is the transverse pelvis axis *Y*_*p*_. The posteroanterior axis *X*_*p*_ is perpendicular to the triangle plane *O*_*p*_ *Y*_*p*_ *Z*_*p*_. The vertical pelvis axis *Z*_*p*_ is orthogonal to *X*_*p*_ and *Y*_*p*_ and belongs to the pelvis triangle plane.

The fixed frame *F*_0_(*OX*_0_ *Y*_0_ *Z*_0_) is affixed to the radiographic set up. *OY*_0_ *Z*_0_ is the frontal anatomical plane and *OX*_0_ *Z*_0_ the sagittal plane. The pelvis is located versus the fixed frame using a translation vector *OO*_*p*_ and a rotation matrix _*p*_^0^ *R*. This last one is obtained from the components of pelvis unit vectors *X*_*p*_, *Y*_*p*_, *Z*_*p*_ projected in frame *F*_0_. This orientation matrix corresponds to a sequence of three successive rotations about the axes of the fixed frame: axial rotation *ψ* about the vertical axis *Z*_0_, lateral flexion *θ* about the posteroanterior axis *X*_0_, and flexion *ϕ* about the transverse axis *Y*_0_.

For purpose of clinical applications, the pelvis orientation represented by the frame *F*_*p*_ is defined by a set of three angles: axial rotation *ψ*_*p*_, lateral flexion *θ*_*p*_, and flexion *ϕ*_*p*_. The geometric pelvis modeling implies, implicitly, that femoral head centers coincide with acetabular cavity centers. Sometimes their positions slightly differ. The transverse pelvis axis *Y*_*p*_ passing through femoral head centers is replaced by the axis connecting the acetabular cavity centers. The pelvis orientation matrix may be weakly modified, as its representing set of orientation angles.

### 3.2. The Sacral Plateau (Figure 2)

The sacral plateau center has been previously located and then used for pelvic geometric modeling. The geometric model of the sacral plateau is based on two hypotheses: the plateau surface is strictly plane, and its anterior edge is a hemiellipsis. The frontal projection of the anterior edge is also a hemiellipsis, of which great half axis is easily identifiable. The perpendicular to this great half axis is assumed to represent the frontal projection of the normal vector to the 3D sacral plateau. The narrow sacral plateau sagittal projection is characterized by a main linear direction. It is supposed that the perpendicular to this main linear direction is the sagittal projection of the normal vector to the 3D plateau. The normal vector 3D position is defined as the intersection between projecting planes (each projecting plane passes through the X-ray source and through the normal vector projection). A set of axis is affixed to the sacral plateau. The center *O*_*s*_ is reconstructed from its two projected images. The unit vector, normal to the sacral plateau, is the vertical axis *Z*_*s*_. The transverse axis *Y*_*s*_ is at the intersection between sacral plateau and horizontal plane *O*_*s*_ *X*_0_ *Y*_0_, *X*_*s*_ is the posteroanterior plateau axis. The sacral plateau is located, versus the fixed frame *F*_0_, using the translation vector *OO*_*s*_ and on orientation matrix _*s*_^0^ *R*. This matrix is determined from the components of *X*_*s*_, *Y*_*s*_, *Z*_*s*_ in frame *F*_0_. The orientation matrix _*s*_^0^ *R* corresponds to a sequence of three successive rotations: axial rotation *ψ*_*s*_ (about *Z*_0_), lateral flexion *θ*_*s*_(*X*_0_), flexion *ϕ*_*s*_(*Y*_0_).

The pelvic morphology is characterized by the position of its articular surfaces versus the pelvis frame. Articular surface locations, versus pelvis, are constant during pelvic movements. The sacral plate relative position, versus pelvis, is defined by both the linear vector *O*_*p*_ *O*_*s*_ projected in frame *F*_*p*_ and by the relative orientation matrix _*s*_^*p*^ *R*, determined from projections of *X*_*s*_, *Y*_*s*_, *Z*_*s*_ unit vectors in frame *F*_*p*_. This matrix corresponds to a sequence of three successive relative rotations: axial rotation *α*_*s*_ (about *Z*_*p*_), lateral flexion *β*_*s*_(*X*_*p*_), flexion *γ*_*s*_(*Y*_*p*_). In real pelvis, the unit vector *Z*_*s*_ perpendicular to the sacral plateau is close to the sagittal plane *O*_*p*_ *X*_*p*_ *Z*_*p*_. So *α*_*s*_ and *β*_*s*_ are small, and *γ*_*s*_ represents the pelvic incidence angle used clinically.

### 3.3. The Acetabular Cavities (Figure 3)

**Figure 1:**
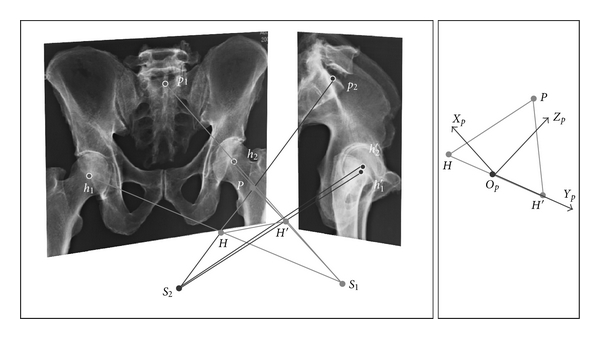
Illustrating the method for locating frames affixed to pelvis from biplanar radiography coupled with geometric modelling. A representative pelvis triangle is extracted from anatomical landmark images and then reconstructed in space from the zones of intersection between couples of projecting rays. A frame *O*_*p*_ *X*_*p*_ *Y*_*p*_ *Z*_*p*_ is affixed to the triangle representing the pelvic bone skeleton.

The two acetabular cavities are treated separately. Each of them, is modeled as a hollow hemisphere, put in the iliac bone. Modeled cavity brims are circular. Frontal and sagittal projections of cavity brims are only detectable on X-ray images. The 3D location of each acetabular cavity is mainly based on the detection of (i) the circular brim centers and (ii) the circular brim normal axes. Acetabular cavities are modeled by their circular edges. Geometric models are projected, radiographically, on frontal and sagittal planes. Their projections are then best fit to real cavity outlines. Circular brims have elliptic frontal and sagittal projections. Real frontal and sagittal brim projections are modeled as ellipses, drawn independently from several recorded points. Centers of projected ellipses are related together, using epipolar plane technique, in order to locate accurately 3D cavity brim centers. Cavity brim circles are centered at this calculated position. Radius values and normal to the circle planes are unknown. Circular cavity brim circles are projected on frontal and sagittal planes. Circle radii and normal directions are chosen so that projections of circular cavity brims best fit corresponding real contours. Frames *F*_*a*_ (*O*_*a*_ *X*_*a*_ *Y*_*a*_ *Z*_*a*_ right acetabulum) and *F*_*a*′_ (*O*_*a*′_ *X*_*a*′_ *Y*_*a*′_ *Z*_*a*′_ left acetabulum) are affixed to acetabular cavities. They are centered at *O*_*a*_ and *O*_*a*′_. *Y*_*a*_ and *Y*_*a*′_ correspond to modeled circular brim axes. Axes *X*_*a*_ and *X*_*a*′_ are determined from the intersection lines between acetabular brim planes and horizontal planes *O*_*a*_ *X*_0_ *Y*_0_ and *O*_*a*′_ *X*_0_ *Y*_0_. *Z*_*a*_ and *Z*_*a*′_ are vertical axes of acetabular cavity edges. Acetabular cavities are located, versus *F*_0_, using translation vectors *OO*_*a*_ and *OO*_*a*′_ and orientation matrices _*a*_^0^ *R* and _*a*′_^0^ *R*. These matrices correspond to sequences of three successive rotations: *ψ*_*a*_ and *ψ*_*a*′_(*X*_0_), *θ*_*a*_ and *θ*_*a*′_(*Y*_0_), *ϕ*_*a*_ and *ϕ*_*a*′_(*Z*_0_). The relative positions of acetabular cavities versus the pelvis frame are described by components in *F*_*p*_ of translation vectors *O*_*p*_ *O*_*a*_ and *O*_*p*_ *O*_*a*′_ and by sequences of three successive rotations *α*_*a*_ and *α*_*a*′_ about *Z*_*p*_, lateral flexion *β*_*a*_ and *β*_*a*′_ about *X*_*p*_, and flexion *γ*_*a*_ and *γ*_*a*′_ about *Y*_*p*_. Sequences of two successive rotations [*α*_*a*_(*Z*_*p*_), *β*_*a*_(*X*_*p*_) and *α*_*a*′_(*Z*_*p*_), *β*_*a*′_(*X*_*p*_)] move *Y*_*p*_ to *Y*_*a*_, and *Y*_*a*′_ respectively. Couples of angular values *α*_*a*_ *β*_*a*_ and *α*_*a*′_ *β*_*a*′_ characterize the orientations of acetabular cavities with respect to the pelvis assumed to be fixed. Asymmetric locations of acetabular cavities, versus pelvis, are displayed. Axes *Y*_*a*_ and *Y*_*a*′_ of acetabular cavities intersect the pelvis sagittal plane *O*_*p*_ *X*_*p*_ *Z*_*p*_ at points *K*_*a*_ and *K*_*a*′_. The relative position of *K*_*a*_ and *K*_*a*′_ allows users to estimate asymmetric positions of acetabuli versus the fixed pelvis. Techniques allowing experimenters to affix frames to pelvis and articular surfaces are summarized in Figure 1 (pelvis), 2 (sacral plateau), 3 (acetabular cavity).

## 4. Results

Pelvic parameters are measured clinically on sagittally X-ray. They are pelvic tilting and pelvic incidence. The pelvic tilting is the angle included between the vertical axis *Z*_0_ and the pelvic vertical axis *Z*_*p*_ connecting the mid-point *O*_*p*_ of femoral head centers to the sacral plateau center *O*_*s*_. The pelvic incidence is included between the pelvic vertical axis *Z*_*p*_ and the axis *Z*_*s*_ perpendicular to the sacral plate. Direct measurements on sagittal X-ray of pelvic tilting and incidence are not accurate when the standing patient pelvis is tilted while radiographed. Angular components calculated from a 3D analysis of pelvis shape and orientation are compared with corresponding values measured on sagittal X-ray.

The 3D approach brings new information: relative positions of acetabular cavities and sacral plateau, so as pelvic asymmetries. This technique has been applied to a significant cohort of standing volunteers: asymptomatic subjects and scoliotic patients. Examples are presented.

Effects of a total hip arthroplasty upon patient pelvic balance are displayed through the comparison between preoperative and postoperative situations.

### 4.1. The Effects of Self-Calibration Procedure

The pelvic tilting and incidence angles measured on sagittal X-ray are not perturbed by errors if two conditions are satisfied: (i) the pelvic frame *O*_*p*_ *X*_*p*_ *Y*_*p*_ *Z*_*p*_ must coincide with the fixed set of axis *OX*_0_ *Y*_0_ *Z*_0_ affixed to the radiographic system (this implies that *Y*_*p*_ and *Y*_0_ are collinear and that the planes of symmetry *O*_*p*_ *X*_*p*_ *Z*_*p*_ and *OX*_0_ *Z*_0_ coincide) and (ii) sacral plateau center and normal vector to sacral plate belong to the pelvis plane of symmetry *O*_*p*_ *X*_*p*_ *Z*_*p*_. If not satisfied, these conditions entail errors in angular measurement on sagittal plane. This can be observed in most sagittal X-rays where the hip axis *Y*_*p*_ is rotated about *Z*_0_ and tilted about *X*_0_. Influences of these pelvic displacements entail errors in plane measurements.

**Figure 4:**
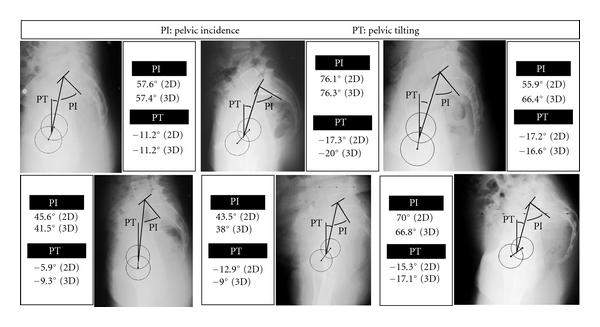
The sagittal X-ray of the standing patient does not represent a pure profile of the pelvis: the hip axis *Y*_*p*_ connecting the femoral head centers is slanted versus the transverse radiographic axis *Y*_0_, and the frame center *O*_*p*_ is not centered versus the radiographic set up. The table presents pelvic tilting and incidence angles measured on sagittal X-ray and the 3D global absolute position of pelvis versus fixed frame and relative position of sacral plate with respect to pelvis (global and relative positions include angular orientations and linear offsets between frame centers).

Six examples of standing pelvis are presented. Pelvic tilting and incidence angles are obtained from 2D measurements and 3D analysis (Figure 4). In some cases, corresponding angular values are close together. In other examples, results differ significantly. The single sagittal radiograph of pelvis cannot explain such differences, contrary to results extracted from 3D analysis.

### 4.2. Main Parameters Describing Orientations of Standing Pelvis and Relative Positions of Articular Surfaces (Sacral Plateau and Acetabular Cavities)

The technique is applied to pelvis of asymptomatic volunteers and of scoliotic patients radiographed in standing stable posture. Biplanar radiography coupled with model best fitting techniques five positions of sets of axis affixed to pelvis and to each articular surface. Pelvis frame is located versus the fixed set of axis using three linear and three angular parameters. Each articular surface set of axis is referred to, with respect to the pelvis frame, by six parameters (three translations and three rotation angles) for a complete location of modeled pelvis and articular surfaces.

A restricted number of parameters have been retained for clinical applications. They are as follows.

- For the pelvis orientation: the three values of rotation angular components (axial rotation, lateral flexion, and flexion).
- For the relative position of articular surfaces versus the pelvis frame: the flexion and lateral flexion components orienting the sacral plate, the axial rotation, and lateral flexion moving the acetabular cavity axis *Y*_*a*_(*Y*_*a*′_) from its initial position *Y*_*p*_.

Indices of pelvic asymmetries have been introduced: an offset describes the linear distance between sacral plateau center *O*_*s*_ and pelvic plane of symmetry *O*_*p*_ *X*_*p*_ *Z*_*p*_, and acetabular cavity axes *Y*_*a*_ and *Y*_*a*′_ intersect the pelvic plane of symmetry at points *K*_*a*_ and *K*_*a*′_. The linear offset *K*_*a*_ *K*_*a*′_ displays the relative asymmetric orientation of acetabular cavities.

**Figure 5:**
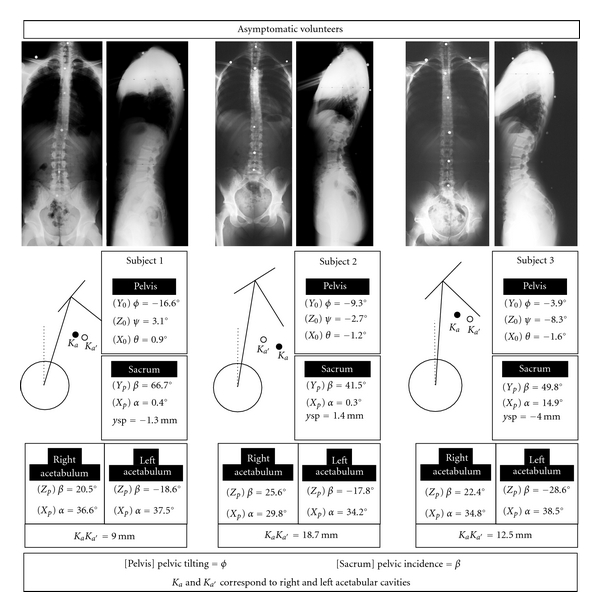
Examples of shapes and orientations of standing pelvis. Parameters describe. (i) 3D pelvic orientation (*ψ*_*p*_, *θ*_*p*_, *ϕ*_*p*_) (ii) The relative position of articular surfaces versus pelvis: flexion *α*_*s*_ and lateral flexion *β*_*s*_ of the sacral plateau, couples of angles (*α*_*a*_, *β*_*a*_) (*α*_*a*′_, *β*_*a*′_) orienting the axis of each acetabular cavity. (iii) The indices of pelvic asymmetry: linear offset ysp of the sacral plateau center, and offset *K*_*a*_ *K*_*a*′_ between cavity axes at level of the pelvic plane of symmetry.

Three examples display the 3D orientation of standing pelvis, as the relative positions of articular surfaces (Figure 5). Pelvic shape asymmetries are related to linear and angular offsets of sacral plateau (subject 1) and to slightly different orientations of acetabular cavity axes (subject 2 and 3).

**Figure 6:**
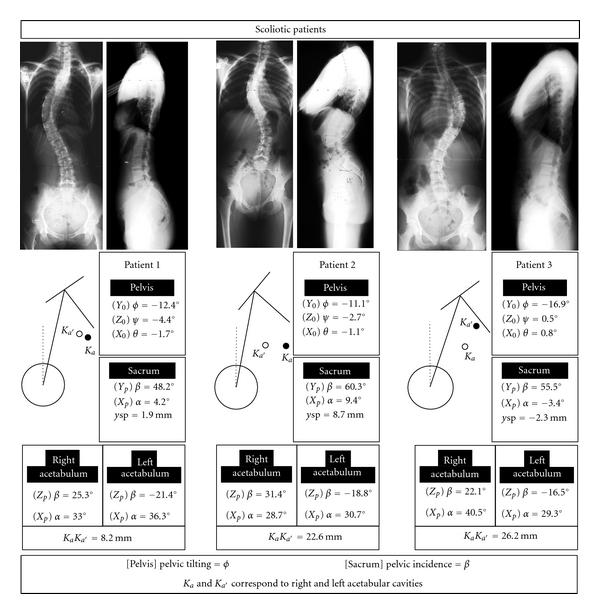
Examples of balanced pelvis of scoliotic patients X-rayed in standing postures. The same main parameters as that retained for asymptomatic subjects are presented.

Three examples show the possible relations between scoliotic spines and pelvis orientation and articular surface positions (Figure 6). Spinal deformities are not strongly related to the standing pelvic balance (case 1). However, asymmetric relative positions of articular surfaces versus pelvis are associated to low back deformities (cases 2 and 3). Clinical studies are in progress for accessing to the influence of pelvic posture and morphology upon spinal deformities.

## 5. Discussion and Conclusion

Our group is involved in joint studies of standing and moving patients. Joint instant features depend on adjacent bone orientations and morphologies. Clinically, 3D bone shapes are often estimated from a unique plane radiograph.

A biplanar radiographic system has been set in a department specialized in deformed spine studies. This system is compound of a standard radiographic set up completed by a rotating platform where patients stand motionless for successive frontal and sagittal exposures. Punctual landmarks and anatomical axes of bones, so as spinal curves, are located in space from their projected images.

Photogrammetric techniques had been first defined for 3D measurements, based on photography, and then adapted to radiography. The main difference between the two applications is that a same X-rayed punctual landmark has scarcely two sharp projections. The couple of projecting rays connecting each X-ray source to each point image do not intersect. Errors reconstructing points, from their projections, are high. They are significantly reduced from using a calibration procedure. Errors decrease from ± 2.5 mm for a point and ± 2° for a direction to ± 1 mm and ± 1°. Different calibration techniques have been proposed. Suh [17] measured errors using an experimental testing including a rigid body. André et al. [18] calculated errors from measurement of X-ray source positions. Labelle et al. [19] calibrated the space, in clinical situation, patients wearing a vest equipped of steel balls X-rayed with them. Berthonnaud et al. [12] used objects of calibration of different sizes.

The biplanar radiographic examination, with successive exposures, is interesting clinically, because it does not require the use of a specific radiographic set up, except a low-price rotating platform. However, errors are due to small patient movements between the two exposures. Thus, error-reconstructing bone points are increased. An autocalibration procedure has been defined for improving accuracy, when reconstructing bone points and axes in standing patients successively radiographed. Several points scatterly distributed are selected. For each of them, couples of projecting rays connecting X-ray source to projected image are drawn. Minimum linear offsets between projecting rays are calculated: they represent the point reconstructing error. X-ray source positions are modified until the mean error extended to all leading points reaches minimum values [14] (about ± 1.5 mm for a point and ± 1.5° for a direction). In clinical situation, the standard calibration is followed by an autocalibration procedure. The autocalibration technique delivers optimal positions of X-ray sources corresponding to the patient specific displacements between successive exposures. Autocalibration effects may be checked clinically from offsets between numerical projections of any punctual landmark with its recorded images.

Long bone anatomical axes are reconstructed from points located along the projections of their axis. 3D positions of anatomical centers and axes are gathered together for defining the personalized bone skeleton. A set of axis is affixed to each bone skeleton. It is based on anatomical landmarks and axis positions. Bone frames are used, for locating corresponding bone versus the radiologic fixed set of axis. Bone morphologic values are calculated from bone skeletons. Clinical applications concerned femurs, tibia [14], shoulder complex [15], and standing spinal geometric structure [13].

Others teams have different approaches: real bone volumes are described from a series of piled up CT cuts. A volumic model of the corresponding bone, issued from a bone atlas, is deformed numerically in order to fit the real bone volume [20, 21]. This technique seems to be more adapted to a realistic representation of bone volumes than to a precise bone frame locating.

Another biplanar radiographic technique has been set for clinical applications [4]. Two radiographic systems, with low-dose radiations, are put together for obtaining simultaneous X-rays of standing patients. Linear radiographic sources are moved simultaneously for describing patient total body. 3D planar slices are piled together for defining real-bone volumes. A registered bone volume model is then fitted to the real one. No calibration procedure is foreseen, even if the patient may move during the testing duration.

The access to pelvic articular surface skeletons and representative frames is impossible, from using uniquely, the 3D reconstruction of punctual landmarks. The pelvis linking sacrum to iliac bone has been modeled as a triangle defined from punctual centers. This triangle represents the pelvis skeleton. A frame is affixed to the pelvis triangle. Articular surfaces are modeled as a plane (sacral plateau) and hemispheres (acetabuli) bounded, respectively, by elliptic edge and circular brims. Edges bounding articular surfaces are only detectable on X-rays. Articular surface centers are first located from their projected images. Then, the modeled sacral plateau and circular acetabular cavity brims are projected on frontal and sagittal X-rays. Frontal and sagittal projections of brim acetabular cavity contours are sometimes hardly identified. A preliminary apprenticeship of experimenters is necessary. A testing involving dry X-rayed pelvis had been set. A dry pelvis is first submitted to a biplanar examination (frontal and sagittal incidences). Then, steel balls are embedded along acetabular cavities edges. Pelvis and balls are then X-rayed in the same conditions. The real projected brim outlines are detected from the positions of X-rayed balls.

The numerical projecting operator is strictly analogous to the radiographic process. The modeled surfaces are slightly shift, till projected modeled brims coincide with real contours represented by several points. When achieved, local frames are affixed to the 3D modeled articular surfaces. Global pelvis set of axis and articular surface frames are first located versus the radiographic frame *F*_0_. Each set of axis is located, versus *F*_0_, using a translation vector defining the frame center position, and an orientation matrix. This last one is expressed as a sequence of three successive simple rotations about axes of the fixed frame: axial rotation *ψ* about *Z*_0_, lateral flexion *θ*(*X*_0_), and flexion *ϕ*(*Y*_0_). This sequence has been preferred to the Euler one, because this last one introduces rotations about moving axes. Thus, results obtained testing different patients are not comparable. The three rotation angles (*ψ*_*p*_, *θ*_*p*_, *ϕ*_*p*_), orienting the pelvis, have clinical meaning. Angles *ψ*_*p*_ and *θ*_*p*_ are respectively: axial rotation and lateral flexion, moving the transverse fixed axis *Y*_0_, to the transverse pelvic axis *Y*_*p*_ (connecting the femoral head centers). The pelvis flexion *ϕ*_*p*_ corresponds to the pelvic tilting angle usually measured on the sagittal X-ray. Articular surface frames are then located with respect to the pelvis. Each articular surface frame is located, versus the pelvis frame, using a translation vector locating the surface center and an orientation matrix. This last one is described by a sequence of three successive rotations about pelvis axes: axial rotation *α* about *Z*_*p*_ (vertical pelvis axis), lateral flexion *β* about *X*_*p*_ (posteroanterior pelvis axis), flexion *γ* about *Y*_*p*_ (transverse pelvis axis). Angles *α*_*s*_, *β*_*s*_, *γ*_*s*_ characterize the sacral plateau orientations, *α*_*s*_ and *β*_*s*_ move the transverse pelvis axis *Y*_*p*_ to *Y*_*s*_ (transverse sacral plateau axis). The sacral plateau flexion angle *γ*_*s*_ corresponds to the pelvic incidence angle measured clinically on sagittal X-ray. Each acetabular cavity is located, versus the pelvis frame, using a translation vector joining pelvic center to acetabular cavity center. In asymptomatic subjects, acetabular cavity centers are close to femoral head centers. Axial rotation *α*_*a*_ and lateral flexion *β*_*a*_ move the transverse axis *Y*_0_ to the normal axis *Y*_*a*_ of the circular acetabular cavity brim.

Linear and angular parameters, describing the relative position of articular surfaces, versus pelvis frame, represent the pelvis morphology. The new approach associates biplanar radiographic examination with elementary modeling of articular surfaces. These last ones are first represented by their modeled edges. Then, the modeled bounded articular surfaces are projected on both X-rays. At last, modeled and real brim projections are fitted together for accessing the optimal location of local articular surfaces and affixed frames.

The chapter results show different clinical applications of the new technique. The first one concerns the relation, in asymptomatic standing subjects, between orientation and pelvis morphology, related to the relative positions of articular surfaces. The second one extends the study to scoliotic patients.

## Figures and Tables

**Figure 2:**
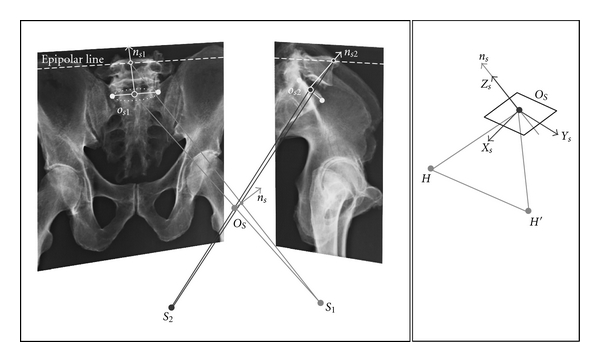
Illustrating the method for locating frames affixed to sacral plateau from biplanar radiography coupled with geometric modelling. The sacral plateau is modelled as a plane bounded by an elliptic brim. Parts of brim projections are shaped as real and flattened ellipses. Sacral plateau center and normal vector projections are identified on each projected brim. Brim center *O*_*s*_ and normal vector *n*_*s*_ are 3D located from using photogrammetric techniques.

**Figure 3:**
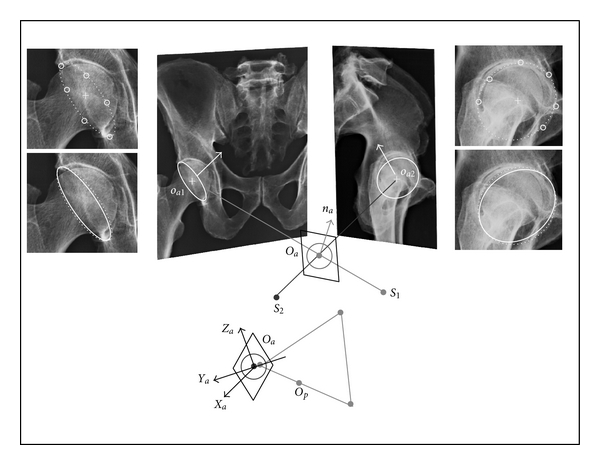
Illustrating the method for locating frames affixed to acetabular cavities from biplanar radiography coupled with geometric modelling. Each acetabular cavity is modelled as an hemisphere bounded by a circular brim. The modelled circular brim of center *O*_*a*_ and axis *n*_*a*_ is projected in frontal and sagittal X-rays. Elliptic projections of circular brim must coincide with the real brim contours. These last ones are determined from landmarks recorded along the contour projections. Real brim images and 3D modelled brim projections are then best fitted.

## References

[1] Hiramoto Y (2000). Morpho-metrical features of the pelvis in standing posture. *Kaibogaku Zasshi*.

[2] Nishihara S, Sugano N, Nishii T, Ohzono K, Yoshikawa H (2003). Measurements of pelvic flexion angle using three-dimensional computed tomography. *Clinical Orthopaedics and Related Research*.

[3] Foroughi P, Song D, Chintalapani G, Taylor RH, Fichtinger G (2008). Localization of pelvic anatomical coordinate system using US/atlas registration for total hip replacement. *Medical Image Computing and Computer-Assisted Intervention*.

[4] Dubousset J, Charpak G, Dorion I (2005). A new 2D and 3D imaging approach to musculo-skeletal physiology and pathology with low-dose radiation and the standing position: the EOS system. *Bulletin de l’Academie Nationale de Medecine*.

[5] D’Lima DD, Urquhart AG, Buehler KO, Walker RH, Colwell CW (2000). The effect of the orientation of the acetabular and femoral components on the range of motion of the hip at different head-neck ratios. *Journal of Bone and Joint Surgery A*.

[6] Seki M, Yuasa N, Ohkuni K (1998). Analysis of optimal range of socket orientations in total hip arthroplasty with use of computer-aided design simulation. *Journal of Orthopaedic Research*.

[7] Laffargue P, Pinoit Y, Tabutin J, Giraud F, Puget J, Migaud H (2006). Computer-assisted positioning of the acetabular cup for total hip arthroplasty based on joint kinematics without prior imaging: preliminary results with computed tomographic assessment. *Revue de Chirurgie Orthopedique et Reparatrice de l’Appareil Moteur*.

[8] Sugano N, Sasama T, Sato Y (2001). Accuracy evaluation of surface-based registration methods in a computer navigation system for hip surgery performed through a posterolateral approach. *Computer Aided Surgery*.

[9] Steppacher SD, Tannast M, Zheng G (2009). Validation of a new method for determination of cup orientation in THA. *Journal of Orthopaedic Research*.

[10] Berthonnaud E, Remy D, Moyen B, Carillon Y, Dimnet J (1998). Inferior limb stereoradiography: technique and applications in clinical practice. *Journal of Biomechanics*.

[11] Liao R, Luc D, Sun Y, Kirchberg K (2010). 3-D reconstruction of the coronary artery tree from multiple views of a rotational X-ray angiography. *International Journal of Cardiovascular Imaging*.

[12] Berthonnaud E, Moyen B, Dimnet J (2000). Stereoradiographic techniques applied to three-dimensional clinical measurements. *Automedica*.

[13] Berthonnaud E, Hilmi R, Dimnet J (2009). Personalized models of bones based on radiographic photogrammetry. *Surgical and Radiologic Anatomy*.

[14] Berthonnaud E, Chotel F, Dimnet J (2002). The anatomic patterns of the lower limb from three-dimensional radiographic reconstruction of bones (3drrb). *ITBM-RBM*.

[15] Berthonnaud E, Herzberg G, Zhao KD, An KN, Dimnet J (2005). Three-dimensional in vivo displacements of the shoulder complex from biplanar radiography. *Surgical and Radiologic Anatomy*.

[16] Berthonnaud E, Dimnet J, Hilmi R (2009). Classification of pelvic and spinal postural patterns in upright position. Specific cases of scoliotic patients. *Computerized Medical Imaging and Graphics*.

[17] Suh CH (1974). The fundamentals of computer aided X ray analysis of the spine. *Journal of Biomechanics*.

[18] André B, Dansereau J, Labelle H (1994). Optimized vertical stereo base radiographic setup for the clinical three-dimensional reconstruction of the human spine. *Journal of Biomechanics*.

[19] Labelle H, Dansereau J, Bellefleur C, Jequier JC (1995). Variability of geometric measurements from three-dimensional reconstructions of scoliotic spines and rib cages. *European Spine Journal*.

[20] Nishii T, Sugano N, Miki H, Koyama T, Takao M, Yoshikawa H (2004). Influence of component positions on dislocation: computed tomographic evaluations in a consecutive series of total hip arthroplasty. *Journal of Arthroplasty*.

[21] Argenson J-N, Ryembault E, Flecher X, Brassart N, Parratte S, Aubaniac J-M (2005). Three-dimensional anatomy of the hip in osteoarthritis after developmental dysplasia. *Journal of Bone and Joint Surgery B*.

